# How to Sit

**Published:** 1854-04

**Authors:** 


### HOW TO SIT.

All consumptive people, and all afflicted with spinal deformities, sit habitually crooked, in one or more curves of the body. There was a time in all these when the body had its natural erectness, when there was the first departure on the road to death. The make of our chairs, especially that great barbarism, the unwieldy and disease-engendering rocking chair, favors these diseases, and undoubtedly, in some instances, leads to bodily habits which originate the ailments just named, to say nothing of piles, fistula, and the like. The painful or sore feeling which many are troubled with incessantly for years, at the extremity of the backbone, is the result of sitting in such a position that it rests upon the seat of the chair, at a point several inches forward of the chair back. A physiological chair, one which shall promote the health and preserve the human form erect and manly as our Maker made it, should have the back straight, at right-angles with the seat, the seat itself not being over eight inches deep. A chair of this kind will do more towards correcting the lounging habits of our youth than multitudes of parental lecturings, for then if they are seated at all they must sit erect, otherwise there is no seat-hold.

In partial connection with this subject, Dr. Potter said, in a recent address at Albany, on

### AMERICAN MANNERS.

"I am a little afraid that a great many people in this country are rather too prone to undervalue this part of education. Certainly we have no admiration for anything finical or affected in manners. We do not want the manners of a village dancing school. But genuine good breeding, genteel manners, ease, modesty and propriety of bearing, we do exceedingly value. When shall we cease to be described as a spitting nation? as a lounging people? When shall we cease to be known by our slovenly speech, by our sitting with our feet higher than our heads? During an excursion of several months in Europe last year, I met hundreds of English at home, and on the continent in every situation. I never saw one spit. I cannot remember that I ever saw any one, however fatigued, lounging or sitting in any unbecoming manner. So long as the State shall feel itself obliged to provide "spittoons" for its legislative halls—so long as the directors of our railroads shall find occasion to put inside of their carriages printed requests to the passengers to "use the spittoons and not the floor, and not to put their feet upon the seats"—so long as we shall continue to fill our conversation and our political harangues with the slang of the fish market, let us not be surprised, nor angry, if foreigners sometimes make themselves witty at our expense. And in the mean time let all those who are intrusted with the care of the young, use their utmost efforts to correct these national barbarisms, and to form the manners of the rising generation after a model more elevated and more refined."

